# Scoring System for Identification of “Survival Advantage” after Successful Percutaneous Coronary Intervention in Patients with Chronic Total Occlusion

**DOI:** 10.3390/jcm9051319

**Published:** 2020-05-02

**Authors:** Tatsuya Nakachi, Shun Kohsaka, Masahisa Yamane, Toshiya Muramatsu, Atsunori Okamura, Yoshifumi Kashima, Shunsuke Matsuno, Masami Sakurada, Yoshitane Seino, Maoto Habara

**Affiliations:** 1 Department of Cardiology, Kanagawa Prefectural Ashigarakami Hospital, 866-1 Matsudasoryo, Matsuda-machi, Ashigarakami-gun, Kanagawa 258-0003, Japan; 2 Department of Cardiology, Keio University School of Medicine, 35 Shinanomachi, Shinjuku-ku, Tokyo 160-8582, Japan; sk@keio.jp; 3 Cardiology Department, Saitama Sekishinkai Hospital, 2-37-20 Irumagawa, Sayama, Saitama 350-1305, Japan; masahisayamane3@gmail.com; 4 Cardiovascular Center, Tokyo General Hospital, 3-15-2 Egota, Nakano-ku, Tokyo 165-8906, Japan; t-mura@tj8.so-net.ne.jp; 5 Division of Cardiology, Sakurabashi Watanabe Hospital, 2-4-32 Umeda, Kita-ku, Osaka 530-0001, Japan; a_okamura@watanabe-hsp.or.jp; 6 Division of Cardiology, Sapporo Cardiovascular Clinic, 8-1, Kita 49-jo Higashi 16-chome, Higashi-ku, Hokkaido 007-0849, Japan; rotacruisingmotion@gmail.com; 7 Department of Cardiovascular Medicine, The Cardiovascular Institute, 3-2-19 Nishiazabu, Minato-ku, Tokyo 106-0031, Japan; matsuno@cvi.or.jp; 8 Department of Cardiology, Tokorozawa Heart Center, 2-61-11, Kamiarai, Tokorozawa, Saitama 359-1142, Japan; masami-saku@mh.point.ne.jp; 9 Cardiology and Vascular Medicine, Hoshi General Hospital, 159-1 Mukaigawara, Koriyama, Fukushima 963-8501, Japan; y.seino@f8.dion.ne.jp; 10 Department of Cardiology, Toyohashi Heart Center, 21-1 Gobudori, Oyama-cho, Toyohashi, Aichi 441-8530, Japan; habara@heart-center.or.jp

**Keywords:** chronic total coronary occlusion, stable ischemic heart disease, percutaneous coronary intervention, coronary revascularization, follow-up study, outcomes

## Abstract

Background: Percutaneous coronary intervention (PCI) is widely used in patients with chronic total occlusion (CTO), but its benefit in improving long-term outcomes is controversial. We aimed to develop a prediction score for grading “survival advantage” conferred by successful results of CTO-PCI and a scoring system for prediction of the influence of CTO-PCI results on major adverse cardiac and cerebrovascular events (MACCEs). Methods: Follow-up data of 2625 patients who underwent CTO-PCI at 65 Japanese centers were analyzed. An integer scoring system was developed by including statistical effect modifiers on the association between successful CTO-PCI and one-year mortality. Results: Follow-up at 12 months was completed in 2034 patients. During follow-up, 76 deaths (3.7%) occurred. Patients with successful CTO-PCI had a better one-year survival than patients with failed CTO-PCI (log rank *P* = 0.016). Effect modifiers for the association between successful procedure and one-year mortality included diabetes (*P* interaction = 0.043), multivessel disease (*P* interaction = 0.175), Canadian Cardiovascular Society class ≥2 (*P* interaction = 0.088), and prior myocardial infarction (MI) (*P* interaction = 0.117). Each component was assigned a single point and summed to develop the scoring system. The patients were then categorized to specify the prediction of survival advantage by successful PCI: ≤2 (normal) and ≥3 (distinct). The differences in one-year mortality between patients with successful and failed treatment were −0.7% and 11.3% for normal and distinct score categories, respectively. In the scoring system for MACCE, score components were prior MI (*P* interaction = 0.19), left anterior descending artery (LAD)-CTO (*P* interaction = 0.079), and reattempt of CTO-PCI (*P* interaction = 0.18). The differences in one-year MACCEs between successful and failed patients for each score category (0, 1, and ≥2) were −1.7%, 7.5%, and 15.1%, respectively. Conclusions: The novel scoring system assessing the advantage of successful PCI can be easily applied in patients with CTO. It is a valid instrument for clinical decision-making while assessing the survival advantage of CTO-PCI and the influence of procedural results on MACCEs.

## 1. Introduction

Continuous technological evolution [1], integration of techniques based on histopathological assessment [2], and development of treatment algorithms [3,4] have improved the success rate of percutaneous coronary intervention (PCI) for chronic total occlusion (CTO) worldwide. Numerous scoring systems have been developed to predict the technical success of CTO-PCI [5,6,7,8], and several reports have validated their accuracy [9]. However, whether successful CTO-PCI would lead to universal “survival advantage” is unclear [10].

Several important prognostic factors, such as reduced left ventricular ejection fraction [11], anemia [12], and hemodialysis [13], have been identified, albeit the prognostic benefit of successful CTO-PCI does not differ according to the presence or absence of these prognostic factors. These factors are helpful in identifying high-risk patients who require close clinical follow-up and/or aggressive risk factor modification. However, their usefulness is limited to decision-making in the preprocedural discussion.

Predictive prognostic advantage dependent on the successful results of CTO-PCI is more appropriate to guide clinical decision-making, not only in the preprocedural discussion but also during the procedure, by indicating the necessity of successful results of the procedure. At present, additional information is required in the preprocedural discussion to assess the prognostic advantage of successful CTO-PCI. Therefore, in this study, we aimed to establish a prediction model for grading survival advantage conferred by the successful results of CTO-PCI. We included effect modifiers on the association between successful procedure and one-year mortality. In addition, we evaluated the performance of this new score and the Multicenter CTO Registry in Japan (J-CTO) score [5], the most widely applied and accepted score, for comparison of its performance. Finally, we developed a scoring system to predict the influence of CTO-PCI results on major adverse cardiac and cerebrovascular events (MACCEs) using a similar strategy.

## 2. Materials and Methods

### 2.1. Study Population

The Retrograde Summit registry is a multicenter, prospective, nonrandomized registry of patients who underwent CTO-PCI at 65 Japanese centers between January 2012 and December 2015. The indication of CTO-PCI or bypass grafting was determined by discussion among the heart team of each institution. Selection of procedural strategy was at the operator’s discretion. The inclusion and exclusion criteria of the registry are described elsewhere [1]. A total of 2625 patients with follow-up data were included in the analyses.

Baseline patient characteristics, procedural details, and clinical outcomes were recorded. Standard definitions of all patient-related variables and clinical diagnoses were used. The techniques used in the procedure have been described previously [1]. There was no centralized event adjudication and core laboratory assessment. All clinical events were reported by each operator of CTO-PCI. This study was approved by the review board of each institution, and written informed consent was obtained from all patients.

### 2.2. Definitions

CTO was defined as complete occlusion with thrombolysis in myocardial infarction (TIMI) grade 0 antegrade flow through the affected segment for >3 months in the opinion of the operator on the basis of clinical features, angiographic features, and/or previous imaging results. Diabetes mellitus (DM) was diagnosed by each physician based on the diagnostic criteria of the Japan Diabetes Society [14]. The estimated glomerular filtration rate was calculated by the modification of diet in renal disease formula [15]. Left ventricular ejection fraction was measured using either contrast left ventriculography or echocardiography. The Canadian Cardiovascular Society (CCS) classification was used to gauge angina severity. Peripheral artery disease was regarded as present when carotid, aortic, or other peripheral artery disease was being treated or the patient was scheduled for surgical or endovascular intervention. Multivessel disease (MVD) was defined as stenosis ≥50% in at least 2 of the 3 major epicardial coronary arteries that the operator deemed to require revascularization. Angiographic morphology of the entry point was classified as “blunt” if the occluded segment did not end in a funnel-tapered form. Lesion calcification was defined as the presence of calcification within the CTO segment, as described elsewhere [5]. Lesion bending was defined as at least one bend of >45° assessed using angiography throughout the occluded segment. Occlusion length was categorized as either <20 or ≥20 mm. The collateral connection grade was classified as previously reported [16]. The J-CTO score was calculated as described by Morino et al. [5] A procedure was defined as “retrograde” if an attempt was made at wiring through collateral arteries; otherwise, it was classified as “antegrade-only”. Procedural success was defined as crossing of a completely occluded lesion with both a guidewire and balloon, resulting in successful dilation of the occluded artery, and restoration of TIMI grade 3 antegrade flow with <50% residual stenosis on final angiography, with no in-hospital MACCEs. In-hospital MACCEs were defined as any of the following adverse events before hospital discharge: all-cause death, myocardial infarction (MI), symptomatic cerebrovascular disease, heart failure, emergency coronary artery bypass grafting (CABG), and emergency target vessel revascularization. MI was defined as an increase in the creatine kinase level to more than two times the upper limit of the normal value.

### 2.3. Outcomes

Clinical information after discharge was obtained from hospital records and via telephone interviews of patients. Of the two scoring systems to be developed for the assessment of the indication of CTO-PCI, the primary endpoint was all-cause mortality for the first one. For the second scoring system, it was MACCEs defined as all-cause death, nonfatal MI, stroke, and target lesion revascularization, including PCI and CABG. When a patient experienced more than one event, the first event was included in the analysis. When at least two events occurred simultaneously, the events were selected in the order of death > nonfatal MI > stroke > target lesion revascularization. Follow-up was censored at month of last follow-up or at 1 year, whichever came first.

### 2.4. Statistical Analysis

Data were statistically analyzed using SPSS Statistics version 24 (IBM, Armonk, NY, USA) and Medcalc 11.1 statistical program (Medcalc, Gent, Belgium). Continuous values are expressed as mean ± standard deviation. Categorical data are presented as frequencies and percentages. Normality was evaluated with the Shapiro–Wilk test. Normally distributed values were compared using an unpaired *t*-test, and non-normally distributed values were compared by the Kruskal–Wallis rank test or the Mann–Whitney *U* test. Chi-squared test or Fisher’s exact test was performed to compare categorical data. Cumulative survival analyses were performed with Kaplan–Meier curve, and difference between curves was assessed by log-rank test.

A comprehensive analysis was performed to develop a scoring system to predict the survival advantage conferred by the successful result of CTO-PCI. Score components were composed of effect modifiers on the association between successful procedure and one-year mortality. Cox proportional hazard models were used to determine the association between procedural results of CTO-PCI and one-year all-cause mortality. However, when taking early hazard into consideration, Cox proportional hazard models might be difficult to be applied. Therefore, the two-way analysis of variance was also performed to extract effect modifiers on the association between CTO-PCI results and one-year mortality. To extract score components among clinical and angiographic characteristics, effect modification of patient characteristics on these associations was determined using interaction terms (patient characteristics × procedural results of CTO-PCI). The effect modifiers with statistical significance or trends on the association between successful CTO-PCI and one-year mortality in the Cox proportional hazard models were assigned 1 point. They were summed to develop the scoring system grading the difference in one-year mortality between patients with successful and failed CTO-PCI. Receiver operating characteristic curves for the scoring systems were created to assess the predictive ability of procedural success.

## 3. Results

### 3.1. Patient Characteristics

The procedural success rate of CTO-PCI in the study patients was 88.8%. Patient characteristics are shown in Table 1. Patients with failed CTO-PCI had more cardiovascular comorbidities, such as hypertension and higher serum creatinine level, than those with successful procedure. A higher prevalence of prior CABG and hemodialysis was also observed in failed patients.

With regard to angiographic characteristics, the CTO target vessel was more likely to be the left anterior descending artery (LAD) in patients with successful CTO-PCI. The CTO lesion in failed patients had more advanced angiographic findings, such as blunt stump, lesion bending, and occlusion length ≥20 mm. Reattempted CTO-PCI was more frequently found in patients with failed CTO-PCI. J-CTO score was higher in failed patients (2.24 ± 1.06 vs. 1.90 ± 0.99, *P* < 0.001).

Procedural characteristics differed between patients with successful and failed CTO-PCI, with a smaller radiation dose (4534 ± 4408 vs. 6175 ± 4661 mGy, *P* < 0.001), smaller contrast dose (214 ± 99 vs. 237 ± 119 mL, *P* < 0.001), shorter fluoroscopy time (66 ± 45 vs. 96 ± 52 minutes, *P* < 0.001), shorter procedure time (149 ± 86 vs. 196 ± 94 min, *P* < 0.001), and lower frequency of retrograde procedure (29% vs. 51%, *P* < 0.001) in patients with successful CTO-PCI.

With regard to periprocedural complications, patients with failed CTO-PCI had significantly higher rates of periprocedural MI, symptomatic cerebrovascular disease, coronary perforation, and access site bleeding.

### 3.2. Development of a Scoring System

**Figure 1:**
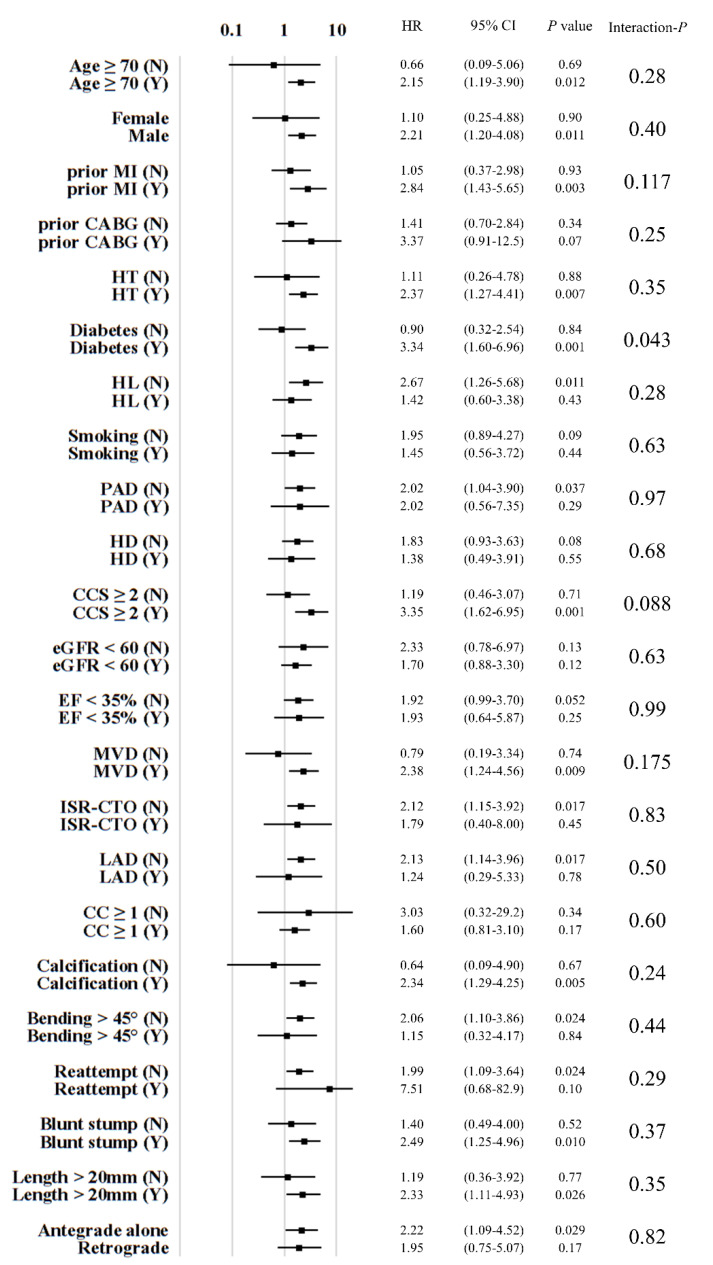
Stratified analysis of hazard ratios for procedural results of CTO-PCI and one-year mortality. Abbreviations: CABG, coronary artery bypass grafting; CC, collateral connection; CCS, Canadian Cardiovascular Society; CI, confidence interval; eGFR, estimated glomerular filtration rate; EF, ejection fraction; HD, hemodialysis; HL, hyperlipidemia; HR, hazard ratio; HT, hypertension; ISR, in-stent restenosis; LAD, left anterior descending artery; MI, myocardial infarction; MVD, multivessel disease; PAD, peripheral artery disease.

Follow-up at 12 months was completed in 2034 (77%) patients. During the mean follow-up duration, 76 deaths (3.7%) were observed. As shown in Figure 1, the presence or absence of DM (*P* interaction = 0.043) significantly modified the association between successful procedure of CTO-PCI and one-year mortality, with particularly higher risk of all-cause mortality in failed patients with DM (hazard ratio (HR) 3.34; 95% confidence interval (CI), 1.60 to 6.96). We observed a trend toward a particularly high risk of mortality among failed patients with CCS ≥2 (*P* interaction = 0.088), MVD (*P* interaction = 0.175), and prior MI (*P* interaction = 0.117). The association between successful CTO-PCI and mortality was consistent across the prespecified stratified analyses for the variables other than those factors.

**Figure 2:**
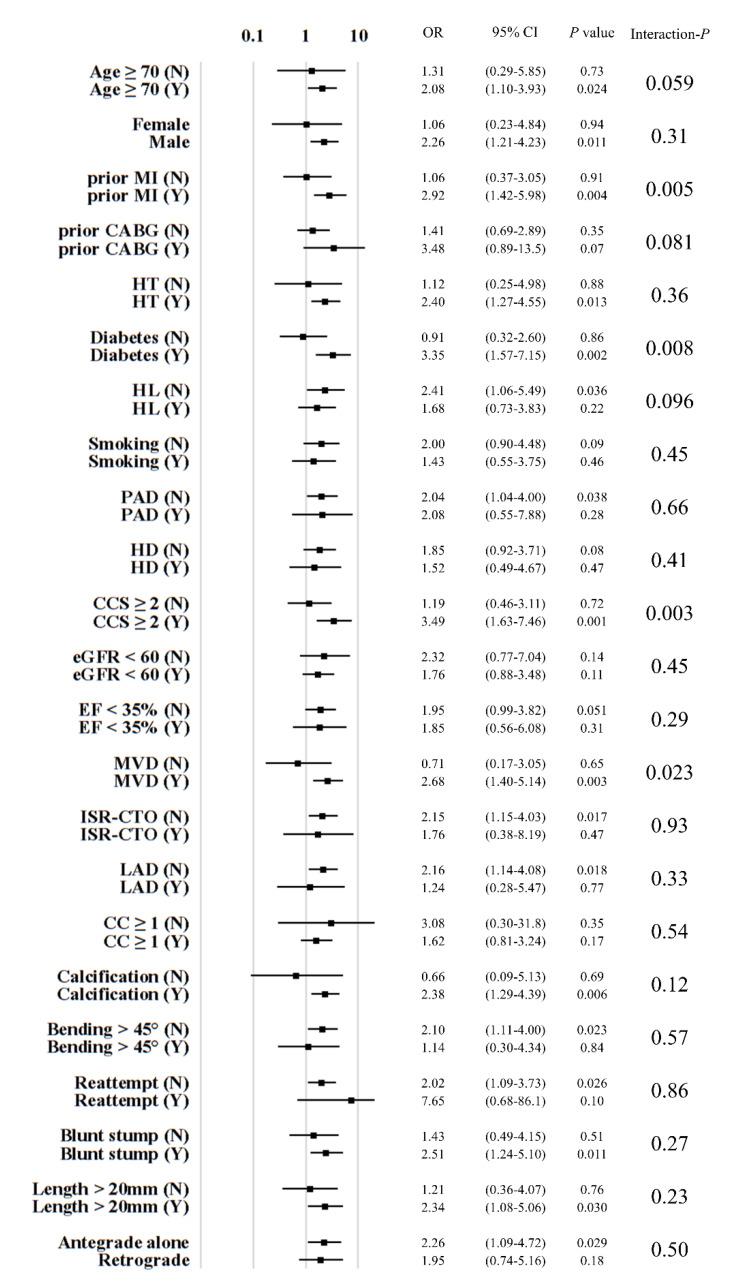
Stratified analysis of odds ratios for procedural results of CTO-PCI and one-year mortality in the two-way analysis of variance. Abbreviations: CABG, coronary artery bypass grafting; CC, collateral connection; CCS, Canadian Cardiovascular Society; CI, confidence interval; eGFR, estimated glomerular filtration rate; EF, ejection fraction; HD, hemodialysis; HL, hyperlipidemia; HT, hypertension; ISR, in-stent restenosis; LAD, left anterior descending artery; MI, myocardial infarction; MVD, multivessel disease; OR, odds ratio; PAD, peripheral artery disease.

On the basis of the statistical significance or trends as effect modifiers on the association between successful CTO-PCI and one-year mortality, DM, CCS ≥2, MVD, and prior MI were included in the HABARA (how aggressively the successful result of CTO-PCI should be achieved by the operator) scoring system for grading the predictive survival advantage conferred by the successful results of CTO-PCI. As shown in Figure 2, the statistically significant effect modifiers in the two-way analysis of variance were the same as the score components extracted in the Cox proportional hazard models. Each of the components was assigned 1 point and summed to develop this scoring system. The HABARA score was categorized to specify the prediction of the survival advantage conferred by successful CTO-PCI: ≤2 (normal) and ≥3 (distinct).

**Figure 3:**
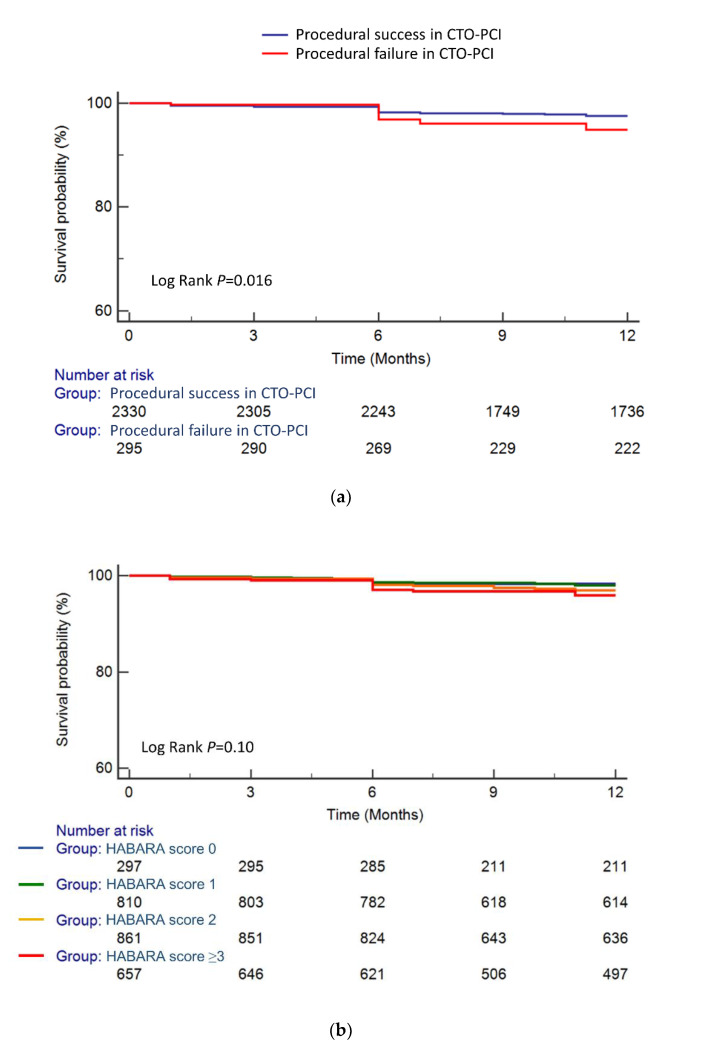

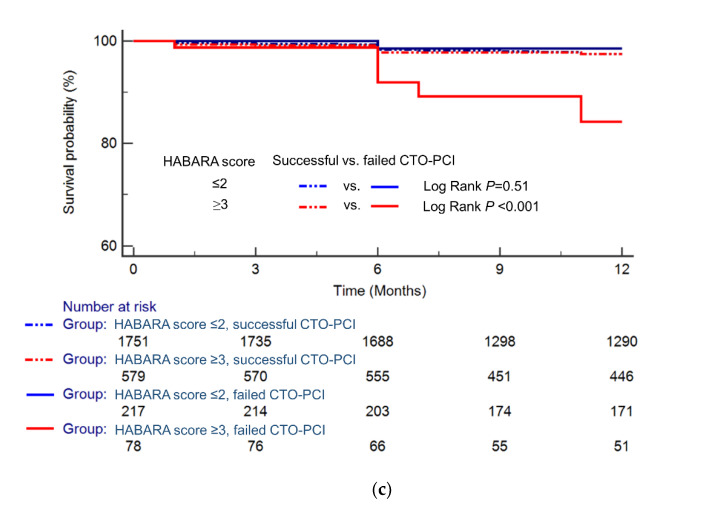
Survival curves after CTO-PCI: (**a**) survival curves after CTO-PCI stratified by procedural results, (**b**) survival curves after CTO-PCI stratified by HABARA score, (**c**) survival curves after CTO-PCI stratified by the HABARA score grade among patients with successful and failed CTO-PCI. Abbreviations: CTO, chronic total occlusion; HABARA, how aggressively the successful result of CTO-PCI should be achieved by the operator; PCI, percutaneous coronary intervention.

**Figure 4:**
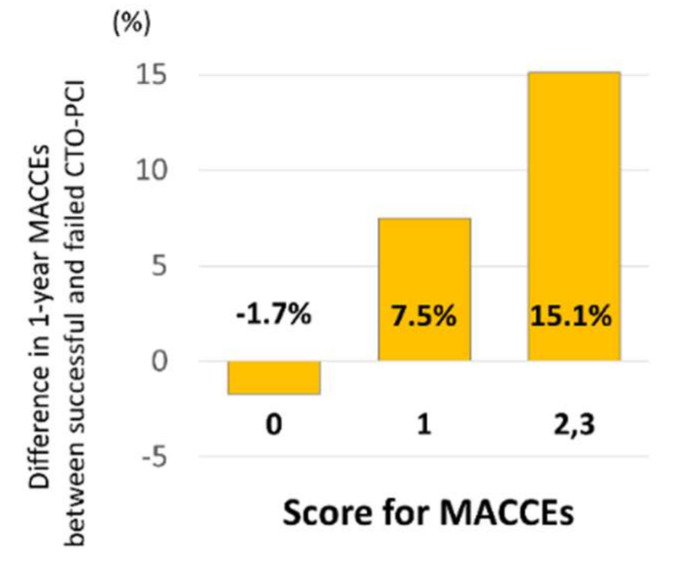
Difference in one-year mortality between patients with successful CTO-PCI and those undergoing failed procedure, stratified by the HABARA score grade. Abbreviations: CTO, chronic total occlusion; HABARA, how aggressively the successful result of CTO-PCI should be achieved by the operator; PCI, percutaneous coronary intervention.

Figure 3a demonstrates that successful percutaneous CTO recanalization was associated with improved one-year survival compared with a failed procedure (log rank *P* = 0.016). Although one-year mortality did not differ according to HABARA score (Figure 3b), successful CTO-PCI was associated with decreased one-year mortality compared to failed procedure among patients with a HABARA score ≥3 (log rank *P* < 0.001) but not among those with HABARA score ≤2 (log rank *P* = 0.51) (Figure 3c). Figure 4 shows that the differences in one-year mortality between successful and failed patients for each of the score categories (normal and distinct) were −0.7% and 11.3%, respectively. Meanwhile, HABARA scores did not differ between successful and failed patients, and the score was not associated, either negatively or positively, with the success rate of CTO-PCI (Table S1).

### 3.3. Depressed Left Ventricular (LV) Function and Involvement of the LAD

Association between each component of the HABARA score and low ejection fraction (EF <35%) or LAD-CTO were additionally investigated (Table S2-1 and Table S2-2). There was perhaps a confounding effect between the variables included in the HABARA score and low EF/target vessel LAD. This was clearer when looking at the direct relationship between the score and these two variables. As seen in Figure S1a, the incidence of low EF (<35%) increased proportionally with the HABARA score (*P* < 0.001). Meanwhile, the incidence of LAD-CTO decreased as the HABARA score increased (*P* < 0.001), as indicated in Figure S1b.

### 3.4. Performance of HABARA Score and J-CTO Score to be Used in Place of Each Other

The performance of the HABARA score and the J-CTO score to be used in place of each other was assessed. Figure S2 demonstrates that the differences in one-year mortality between successful and failed patients for each of the J-CTO score category (easy, intermediate, difficult, and very difficult) were −2.5%, −1.5%, 4.9%, and 2.8%, respectively. As shown in Figure S3, the J-CTO score grade allowed significant discriminatory ability to predict procedural failure of CTO-PCI (area under the curve, 0.59; 95% CI, 0.55 to 0.62; *P* < 0.001), but the HABARA score grade did not (area under the curve, 0.51; 95% CI, 0.47 to 0.54; *P* = 0.66).

### 3.5. Prediction of the Influence of the Results of CTO-PCI on the MACCE Rate

During the follow-up period, 258 MACCEs occurred (60 deaths, 1 nonfatal MI, 13 strokes, and 140 target lesion revascularization in successful patients; 15 deaths, 9 strokes, and 20 target lesion revascularization in failed patients). We applied our scoring system for the prediction of MACCEs (all-cause death, nonfatal myocardial infarction, stroke, and target lesion revascularization, including both PCI and CABG).

**Figure 5:**
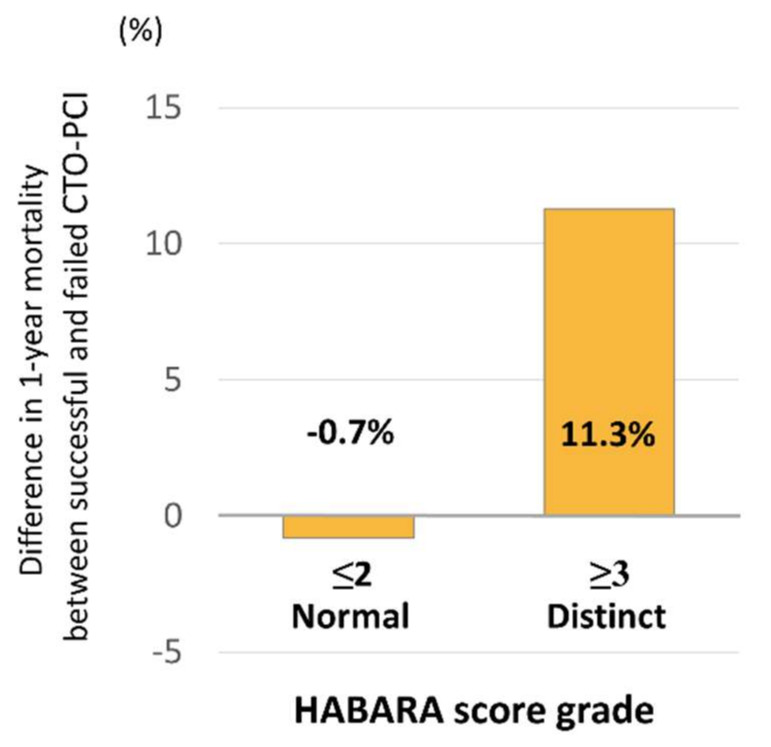
Difference in one-year MACCEs between patients with successful CTO-PCI and those undergoing failed procedure, stratified by the HABARA score grade. Abbreviations: CTO, chronic total occlusion; HABARA, how aggressively the successful result of CTO-PCI should be achieved by the operator; MACCEs, major adverse cardiac and cerebrovascular events; PCI, percutaneous coronary intervention.

Comparison of Figure 5 and Figure 4 shows that the inclusion of events other than death did not affect the ability of the HABARA score to stratify differences in adverse event rates between patients who underwent successful and failed CTO-PCI. Additionally, we developed a second scoring system to predict the influence of the results of CTO-PCI on the MACCE rate.

**Figure 6:**
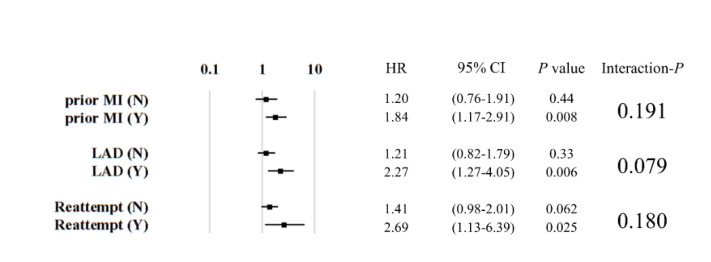
Effect modifiers with statistical trends on the association between successful CTO-PCI and 1-year MACCEs. Abbreviations: CI, confidence interval; LAD, left anterior descending artery; MI, myocardial infarction.

**Figure 7:**
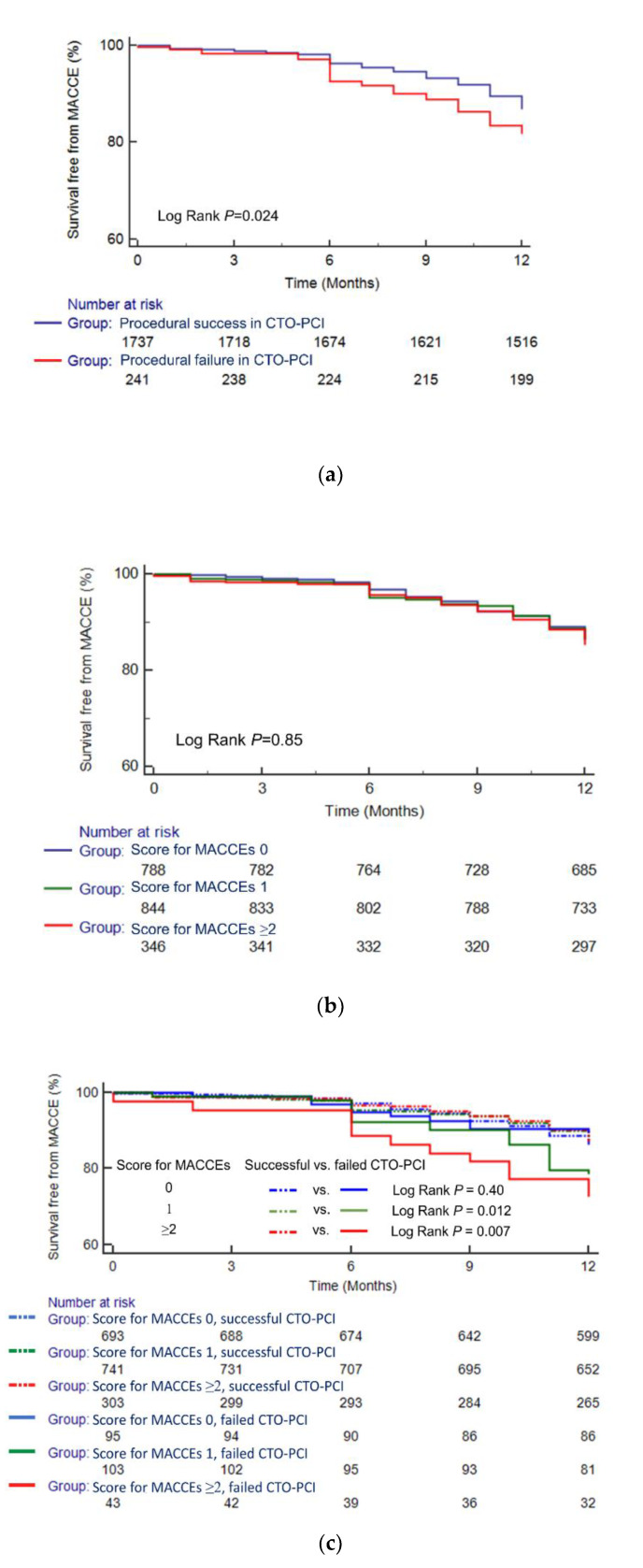
Survival free from MACCE after CTO-PCI: (**a**) survival free from MACCEs stratified by procedural results, (**b**) survival free from MACCEs stratified by procedural results, (**c**) survival free from MACCEs stratified by score for MACCEs among patients with successful and failed CTO-PCI. Abbreviations: CTO, chronic total occlusion; MACCEs, major adverse cardiac and cerebrovascular events; PCI, percutaneous coronary intervention.

**Figure 8:**
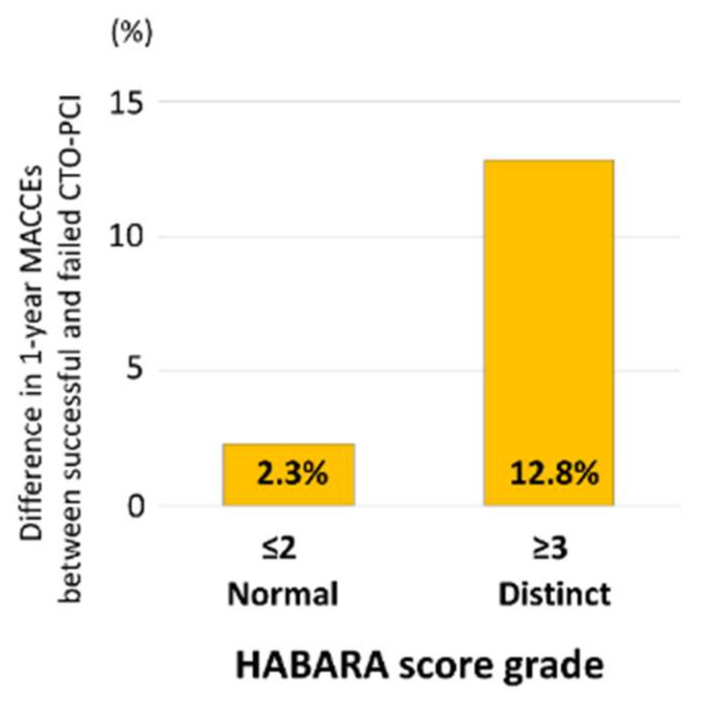
Difference in one-year MACCEs between patients with successful CTO-PCI and those undergoing failed procedure, stratified by the score for MACCEs. Abbreviations: CTO, chronic total occlusion; MACCEs, major adverse cardiac and cerebrovascular events; PCI, percutaneous coronary intervention.

Included variables were prior MI (*P* interaction = 0.19), LAD-CTO (*P* interaction = 0.079), and reattempt of CTO-PCI (*P* interaction = 0.18), with each assigned a single point (Figure 6). The performance of this second scoring system is demonstrated in Figure 7. Patients with failed CTO-PCI had a higher MACCE rate than those with a successful procedure (Figure 7a). Similar to our first scoring system, one-year MACCEs did not differ according to the assigned score (Figure 7b). However, successful CTO-PCI was associated with lower one-year MACCEs than failed procedures among patients with a score for MACCEs of 1 (log rank *P* = 0.012) or ≥2 (log rank *P* = 0.007) but not among those with a score for MACCEs of 0 (log rank *P* = 0.40; Figure 7c). Figure 8 shows that the differences in one-year MACCEs between successful and failed patients for each of the score categories (0, 1, and ≥2) were −1.7%, 7.5%, and 15.1%, respectively.

## 4. Discussion

This study has two major findings. First, the effect modifiers of this association with statistical significance or trends were DM, CCS ≥2, MVD, and prior MI. Second, by including these effect modifiers, we established a scoring model for grading the predicted survival advantage conferred by the successful results of CTO-PCI. This score stratified the difference in one-year mortality between patients with successful and failed CTO-PCI ranging from 0% to 11%. The performance of this novel scoring system and the J-CTO score was limited to be used in place of each other. The HABARA score would likely aid in preprocedural decision-making by indicating the “survival advantage” of successful results rather than predicting the short-term procedural outcome.

### 4.1. Utility of the HABARA Score

Given the maturity of scoring systems to predict successful results of CTO-PCI [5,6,7,8], additional information for optimal patient selection is a compelling need. Patients with CTO are often recognized as high-risk patients with high Synergy between PCI with TAXUS and Cardiac Surgery score [17]. Treatment strategy is not affected much by the prognostic factors, particularly in the Asia Pacific region with lower rates of CABG in comparison with North America and Europe. Therefore, rather than the prognostic factors, the predictive survival advantage conferred by the successful results of the procedure is more clinically important in the preprocedural discussion by indicating the necessity of successful results in index CTO-PCI. Previous reports have shown that the survival advantage of successful CTO-PCI depends on CTO vessel [18,19,20]. However, the results were not consistent, and interaction analyses were not performed to assess the statistical significance of CTO vessel as an effect modifier of the association between procedural results and mortality. Furthermore, compared with procedural results, survival advantage dependent on the successful results of CTO-PCI is much more difficult for physicians to predict on the basis of their experience and impression from daily clinical practice. Hence, we performed a comprehensive analysis to develop a scoring system to predict the survival advantage conferred by the successful result of CTO-PCI by including effect modifiers on the association between successful procedure and one-year mortality.

The applicability of the HABARA score and the J-CTO score, originally developed to predict guidewire crossing within 30 min and the most widely applied score for procedural results, seemed to complement each other. No score component was mutual for the two scoring systems. We suggest that each piece of information should be assessed separately using each of the dedicated scoring systems. The HABARA score identifies the effect of the procedural results of CTO-PCI on all-cause mortality. Accordingly, treatment strategy (the indication for CTO-PCI) should be determined based not only on the HABARA score but also on other scoring systems used to predict procedural results, such as the J-CTO score. Among candidate patients for CTO-PCI with a high HABARA score, those with a high likelihood of procedural success should be highly recommended to undergo the procedure due to expectation of better survival outcomes and those with a low likelihood of success might avoid treatment because of concerns regarding increased mortality. Moreover, this new score is potentially helpful during the procedure, for instance, in clinical decision-making (whether to stop the procedure or to continue by moving on to the next technical option and/or using additional devices even with elevated radiation dose and increased contrast load).

### 4.2. Score Components

On the basis of the statistical trends as effect modifiers, MVD, CCS ≥2, and prior MI were selected as score components in the HABARA score. MVD is associated with adverse outcomes in patients with coronary artery disease [21] and with technically unsuccessful results in patients undergoing CTO recanalization [22]. Despite this, MVD has not been included as a score component among recently developed scoring systems to gauge the likelihood of success in CTO-PCI [5,6,7,8], and no study has identified the prognostic advantage of MVD associated with successful CTO-PCI. MVD is known to impact selection of treatment strategy for patients with CTO, decreasing the likelihood of PCI. This may indicate the specific survival impact of successful results in CTO-PCI referred due to surgical ineligibility. Considering the advances in device and technique, PCI is rapidly becoming a valid option for complex higher-risk (and indicated) patients (CHIP) [23]. The characteristics of patients undergoing PCI in clinical practice in Japan and Western countries differ substantially; Japanese patients tend to have a higher rate of complex lesions and a longer procedure time [24,25]. Our results indicate that with widespread use of PCI in CHIP, MVD may have important clinical implications for predicting long-term outcomes.

Inclusion of CCS ≥2 is in line with prior reports, in which increased mortality [26] and adverse cardiovascular outcomes [27] correlated with angina severity in stable outpatients, and improvements in left ventricular function following CTO-PCI correlated with angina severity [28]. Moreover, a randomized trial recently demonstrated the clinical benefit of CTO-PCI over medical treatment as an improvement in symptom control and quality of life [29]. Taking a bias inherent in randomized trials to include less symptomatic patients into consideration, our results may expand on those findings. Information on whether the distribution of prior MI was in the perfusion territory of the target CTO lesion was not obtained in this registry. Data regarding myocardial distribution of infarction, viability, and ischemic territory evaluated with scintigraphy or cardiovascular magnetic resonance were not routinely collected in the current registry. However, they are required for in-depth discussion of the prognostic benefit conferred by successful CTO-PCI.

DM was included as a component in the HABARA score because of its statistical significance as an effect modifier on the association between successful procedure and all-cause mortality. A previous report [30] showed that the benefits of successful CTO-PCI on long-term cardiac mortality particularly manifest in diabetic patients; however, DM was not a significant effect modifier in the interaction analysis. This discrepancy can be explained by the difference in sample size.

The score components above have been repeatedly shown to be significant predictors of long-term outcomes in revascularized patients (Table S3). Meanwhile, depressed LV function and involvement of the proximal LAD are also important predictors of long-term outcome. However, as shown in Figure 1, both EF <35% and LAD-CTO were not effect modifiers for the association between CTO-PCI results and all-cause mortality in our analysis. Based on the direct relationship between the HABARA score and these two variables indicating confounding effects, inclusion of low EF/LAD-CTO was omitted.

The only mutual component between the two scoring systems developed in the current study was prior MI. Both LAD-CTO and reattempted procedure might indicate the importance of the procedure and high prevalence of revascularization, especially in failed patients.

### 4.3. Limitations

Several limitations should be acknowledged in this study. First, the Retrograde Summit is a prospective real-world single-arm registry and not a randomized clinical trial. As such, the study provides no information on outcomes of patients with CTO without undergoing PCI. The results of observational studies, such as ours, are prone to bias and are, at best, hypothesis-generating. However, appropriately identifying candidates for revascularization procedures remains a critical issue in the management of patients with CTO. Although revascularization of CTO was long believed to improve patients’ prognosis, recent randomized trials, such as the EuroCTO [29] and DECISION-CTO [31], did not support this hypothesis and conversely demonstrated that routine (or prophylactic) revascularization does not improve patients’ survival. Therefore, at present, the key question for CTO lesions is to identify the subset of patients that would benefit from successful revascularization procedures [32]. We believe that this study provides important information to fill this gap. The results of our study cannot fully address whether “patients with a successful procedure do better because of the procedure” or “success is more likely in patients with a better survival expectation”. However, when considered together with previously published reports, the identified variables in our study (DM, MVD, significant symptoms, and prior MI) have been repeatedly shown to be significant predictors of long-term outcomes in revascularized patients (Table S3). On the other hand, when the procedural success rate was compared between patient groups with different scores, it did not correlate (either negatively or positively; Table S1) with the original cohort. Therefore, we believe the former explanation (patients with a successful procedure do better because of the procedure) is more likely than the latter (success is more likely in patients with a better survival expectation). Second, although the primary endpoint of this study was all-cause death, improving survival is not the only potential benefit of CTO-PCI. Improvements also exist in clinical outcomes, such as reduced incidence of MI, reduced need for CABG, improvement in left ventricular systolic function [33], and significant reductions in angina burden after CTO-PCI. These potential benefits may also justify attempted CTO-PCI despite a lack of significant improvements in one-year survival in some patient subsets. Third, data regarding medical therapies and laboratory measurements of lipid profiles, inflammatory markers, and hemoglobin were not obtained. Fourth, no centralized event adjudication and core laboratory assessment were performed. All clinical events were reported by each operator who performed CTO-PCI. Finally, due to high loss of follow-up, the number of patients with CTO was inadequate for application of the holdout validation by splitting the original data into independent derivation and validation sets. Therefore, further study is warranted to evaluate the applicability of the HABARA score in other patient populations. Meanwhile, comparing the components of the HABARA score between patients lost and not lost during follow-up (deaths and survivors), only prior MI significantly differed between the two groups, with a lower rate of prior MI in patients lost during follow-up (40% vs. 34%, *P* = 0.022) (Table S4). Thus, despite the high loss to follow-up, we believe that the main conclusion of our study remains given the minimal differences between the two groups.

## 5. Conclusions

Given the maturity of prediction models for procedural results of CTO-PCI, additional information of the prognostic effect of successful procedure remains a compelling need. We developed a scoring system for grading the predictive survival advantage conferred by the successful results of CTO-PCI. By indicating the necessity of successful results in CTO-PCI, this score is a valid instrument in clinical decision-making.

## Figures and Tables

**Table 1:** Patient characteristics.

	Successful CTO-PCI	Failed CTO-PCI	*P* Value
	(*n* = 2330)	(*n* = 295)	
Clinical characteristics			
Age, years	68 ± 10	69 ± 10	0.22
Male	1950 (84%)	241 (82%)	0.32
Prior MI	861 (38%)	112 (39%)	0.79
Prior CABG	179 (8%)	44 (15%)	<0.001
Hypertension	1806 (78%)	246 (84%)	0.026
Diabetes mellitus	1014 (44%)	128 (44%)	0.93
Hyperlipidemia	1639 (71%)	204 (70%)	0.76
Smoker	1177 (54%)	134 (48%)	0.09
Peripheral vascular disease	273 (13%)	41 (15%)	0.27
Hemodialysis	114 (5%)	33 (12%)	<0.001
CCS 0	755 (34%)	96 (33%)	0.61
CCS I	630 (28%)	92 (32%)	
CCS II	656 (29%)	82 (28%)	
CCS III	144 (6%)	14 (5%)	
CCS IV	51 (2%)	5 (2%)	
Cr, mg/dL	1.4 ± 2.2	1.8 ± 2.6	0.001
eGFR, mL/min/1.73 m^2^	61.9 ± 22.7	59.5 ± 49.3	0.153
LVEF			
<35%	176 (8%)	29 (10%)	0.32
35%–50%	513 (23%)	70 (25%)	
>50%	1523 (69%)	185 (65%)	
Cerebrovascular disease	92 (4%)	17 (6%)	0.14
Angiographic characteristics			
Multivessel disease	1376 (60%)	192 (66%)	0.07
CTO vessel			
Right	1073 (46%)	159 (54%)	0.012
Left anterior descending	745 (32%)	67 (23%)	
Left circumferential	497 (21%)	68 (23%)	
Left main trunk	8 (0.3%)	1 (0.3%)	
In-stent occlusion	360 (16%)	35 (12%)	0.102
Blunt stump	1222 (53%)	185 (64%)	0.001
Lesion calcification	1843 (80%)	239 (83%)	0.242
Lesion bending	174 (8%)	46 (10%)	<0.001
Occlusion length ≥20 mm	988 (51%)	136 (62%)	0.003
Reattempted lesion	200 (9%)	54 (19%)	<0.001
Collateral channel classification			
CC0	123 (6%)	14 (6%)	0.54
CC1	1243 (64%)	161 (68%)	
CC2	575 (30%)	63 (27%)	
J-CTO score	1.90±0.99	2.24±1.06	<0.001
Procedural characteristics			
Air kerma, mGy	4534 ± 4408	6175 ± 4661	<0.001
Procedural time, min	149 ± 86	196 ± 94	<0.001
Fluoroscopy time, min	66 ± 45	96 ± 52	<0.001
Contrast dose, mL	214 ± 99	237 ± 119	<0.001
Retrograde procedure	664 (29%)	149 (51%)	<0.001
Periprocedural complications			
Periprocedural MI	0 (0%)	10 (3%)	<0.001
Symptomatic cerebrovascular disease	0 (0%)	4 (1.4%)	<0.001
Coronary perforation	51 (2.2%)	23 (7.9%)	<0.001
Access site bleeding	8 (0.3%)	5 (1.7%)	0.002
Heart failure	0 (0%)	0 (0%)	-
Emergency CABG	0 (0%)	0 (0%)	-
Emergency TVR	0 (0%)	0 (0%)	

Values are presented as mean ± standard deviation or as numbers (percentages). CABG, coronary artery bypass grafting; CC, collateral channel; CCS, Canadian Cardiovascular Society; Cr, creatinine; CTO, chronic total occlusion; eGFR, estimated glomerular filtration rate; J-CTO, Multicenter CTO Registry in Japan; LVEF, left ventricular ejection fraction; MI, myocardial infarction; PCI, percutaneous coronary intervention; TVR, target vessel revascularization.

## Supplementary Materials

The following are available online at https://www.mdpi.com/2077-0383/9/5/1319/s1, Table S1: A. Average HABARA score in successful and failed CTO-PCI. B. Procedural success rate by HABARA score. Table S2-1: The association between EF < 35% and each component of the HABARA score. Table S2-2: The association between LAD-CTO and each component of the HABARA score. Table S3: List of publications on predictors of long-term outcome amongst revascularized patients. Table S4: Comparison of patient characteristics between patients who completed the 12-month follow-up and those who did not. Figure S1: The association between HABARA score and established predictive factors, such as (a) LVEF < 35% and (b) target vessel LAD. Figure S2: Difference in one-year mortality between patients with successful CTO-PCI and those undergoing failed procedure, stratified by the J-CTO score grade. Figure S3: Comparison of the discriminatory ability of scoring systems to predict procedural failure of CTO-PCI.
