# Supplementary material for: Scoring System for Identification of “Survival Advantage” after Successful Percutaneous Coronary Intervention in Patients with Chronic Total Occlusion
Source: J Clin Med. 2020 May 2;9(5):1319. doi: 10.3390/jcm9051319 (PMC7291306; doi:10.3390/jcm9051319)
Supplement: Supplementary file 1 [file jcm-09-01319-s001.pdf]

Journal of Clinical Medicine

MDPI

Supplementary materials

Table S1. A. Average HABARA score in successful and failed CTO-PCI. **B.** Procedural success rate by HABARA score.

A.

	Successful CTO-PCI (n=2330)	Failed CTO-PCI (n=295)	P value
HABARA Score	1.76±1.05	1.81±1.06	0.48

B.

HABARA Score	0 (n=297)	1 (n=810)	2 (n=861)	3 (n=525)	4 (n=132)	P value
CTO-PCI Success Rate	268 (90.2%)	715 (88.3%)	768 (89.2%)	465 (88.6%)	114 (86.4%)	0.77

Table S2-1. The association between EF<35% and each components of HABARA score.

		**DM**		**CCS≥2**		**MVD**		**Prior MI**	
		(-)	(+)	(-)	(+)	(-)	(+)	(-)	(+)
**EF<35%**	(-)	52.0%	39.9%	57.9%	33.9%	36.9%	54.8%	58.8%	32.9%
	(+)	3.9%	4.3%	4.6%	3.6%	2.4%	5.9%	3.1%	5.2%
		P = 0.014		*P* = 0.07		*P* = 0.001		*P* <0.001	

Table S2-2. The association between LAD-CTO and each components of HABARA score

		**DM**		**CCS≥2**		**MVD**		**Prior MI**	
		(-)	(+)	(-)	(+)	(-)	(+)	(-)	(+)
**LAD-CTO**	(-)	38.5%	30.5%	42.3%	26.7%	24.7%	44.3%	43.5%	25.5%
	(+)	17.4%	13.6%	20.0%	11.0%	14.5%	16.5%	18.2%	12.8%
		P = 0.014		*P* = 0.07		*P* = 0.001		*P* <0.001	

Table S3. List of publications on predictors of long term outcome amongst revascularized patients.

Diabetes mellitus:

- Among patients undergoing angioplasty, compared with non-DM, DM patients had a higher 5-year mortality, MI, and a greater need for additional revascularization. (Circulation 1995;91:979-89)
- Among patients undergoing successful stent replacement for CAD, DM was associated with a higher incidence of death, MI, and reinterventions during 1-year follow-up. (J Am Coll Cardiol 1998;32:1866)
- Subanalysis of SYNTAX study: Mortality was significantly higher among DM vs. non-DM patients after both PCI and CABG treatments. (J Am Coll Cardiol 2010;55:1067-75)
- Credo-Kyoto registry: DM was associated with poorer outcomes in non-HD patients, it no longer had a major impact on outcomes in HD patients. (Circ J 2011;75:1616-25)

Multivessel disease:

- CASS registry: The extent of coronary obstructive disease and the status of LV function are powerful predictors of survival for patients receiving medical treatment. (Circulation 1994;90:2645-57)

Symptoms of CCS ≥2:

- CLARIFY registry (2-year follow-up): In stable CAD patients, angina symptoms but not silent ischemia was associated with an increased risk for adverse cardiovascular outcomes during 2-year follow-up. (JAMA Intern Med 2014;174:1651-59)

Prior myocardial infarction:

- CLARIFY registry (5-year follow-up): Among patients with chronic coronary syndrome, prior MI was a determinant of poor prognosis regardless of angina symptoms. Meanwhile, although angina was associated with a poor prognosis, this was only true in patients with prior MI but not in patients without prior MI. (Eur Heart J 2020;41:347-55)

Table S4. Comparison of patient characteristics between patients who completed the 12-month follow-up and those who did not

	**Patients that completed follow-up (n=2034)**	**Patients lost during the follow-up period (n=591)**	***P* value**
**Clinical characteristics**			
**Age, years**	68±10	68±10	0.68
**Male**	1683 (83%)	508 (85%)	0.35
**Prior MI**	770 (40%)	203 (34%)	**0.022**
**Prior CABG**	167 (8%)	56 (9%)	0.40
**Hypertension**	1581 (79%)	471 (80%)	0.63
**Diabetes mellitus**	899 (45%)	243 (41%)	0.10
**Hyperlipidemia**	1439 (72%)	404 (68%)	0.079
**Smoker**	1012 (53%)	299 (53%)	0.92
**Peripheral vascular disease**	229 (12%)	85 (15%)	0.062
**Hemodialysis**	105 (5%)	42 (7%)	0.10
**CCS 0**	650 (33%)	201 (35%)	0.77
**I**	560 (29%)	162 (28%)	
**II**	573 (29%)	165 (29%)	
**III**	125 (6%)	33 (6%)	
**IV**	40 (2%)	16 (3%)	
**Cr, mg/dL**	1.4±2.3	1.5±2.1	0.12
**eGFR, mL/min/1.73 m^2**	62.0±27.8	60.5±24.1	0.27
**LVEF**			
**<35%**	146 (8%)	59 (10%)	0.10

**35–50%**	457 (24%)	126 (22%)	
**>50%**	1320 (69%)	388 (68%)	
**Cerebrovascular disease**	82 (4%)	27 (5%)	0.61
**Angiographic characteristics**			
**Multivessel disease**	1208 (61%)	360 (61%)	0.90
**CTO vessel**			
**Right**	948 (47%)	284 (48%)	0.84
**Left anterior descending**	628 (31%)	184 (31%)	
**Left circumferential**	439 (22%)	126 (21%)	
**Left main trunk**	8 (0.4%)	1 (0.2%)	
**In-stent occlusion**	281 (14%)	114 (19%)	**0.002**
**Blunt stump**	1093 (54%)	314 (53%)	0.59
**Lesion calcification**	1612 (80%)	470 (80%)	0.76
**Lesion bending**	176 (9%)	44 (7%)	0.31
**Occlusion length ≥20 mm**	890 (52%)	234 (51%)	0.71
**Reattempted lesion**	210 (11%)	44 (8%)	**0.031**
**Collateral channel classification**			
**CC0**	116 (7%)	21 (4%)	**0.033**
**CC1**	1071 (63%)	333 (69%)	
**CC2**	508 (30%)	130 (27%)	
**J-CTO score**	1.96±1.01	1.85±0.96	**0.014**
**Procedural characteristics**			
**Air kerma, mGy**	4685±4498	4831±4361	0.54
**Procedural time, min**	156±91	148±80	0.068
**Fluoroscopy time, min**	71±48	65±42	**0.013**
**Contrast dose, mL**	215±99	222±108	0.18
**Retrograde procedure**	643 (32%)	170 (28%)	0.13

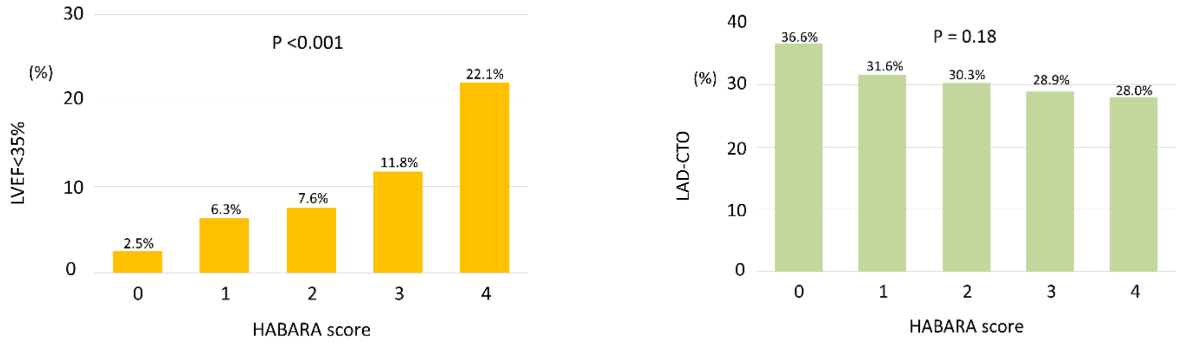

(**a**) (**b**)

Figure S1. The association between HABARA score and established predictive factors such as (a) LVEF<35% and (b) target vessel LAD. Abbreviations: CTO, chronic total occlusion; HABARA, how aggressively the successful result of CTO-PCI should be achieved by the operator; LAD, left anterior descending artery; LVEF, left ventricular ejection fraction.

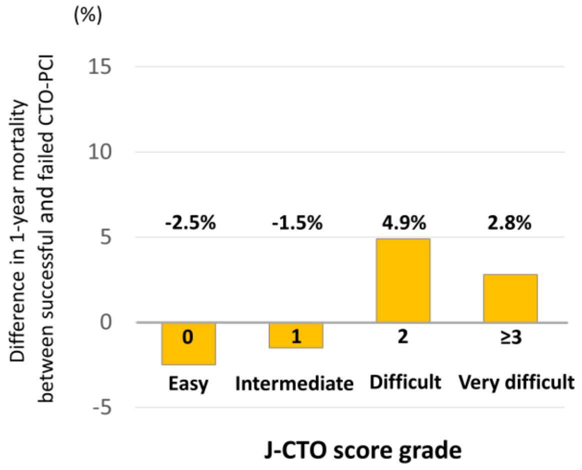

Figure S2. Difference in 1-year mortality between patients with successful CTO-PCI and those undergoing failed procedure, stratified by the J-CTO score grade. Abbreviations: CTO, chronic total occlusion; J-CTO, Multicenter CTO Registry in Japan; PCI, percutaneous coronary intervention.

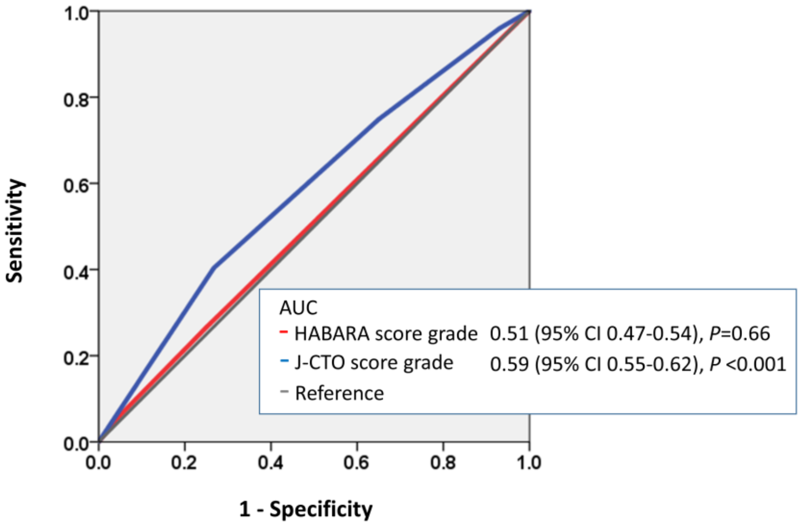

Figure S3. Comparison of the discriminatory ability of scoring systems to predict procedural failure of CTO-PCI. Abbreviations: CTO, chronic total occlusion; HABARA, how aggressively the successful result of CTO-PCI should be achieved by the operator; J-CTO, Multicenter CTO Registry in Japan; PCI, percutaneous coronary intervention.
